# Finite element analysis of dental structures: the role of mandibular kinematics and model complexity

**DOI:** 10.3389/fdmed.2024.1461909

**Published:** 2024-09-12

**Authors:** Canan Özcan, Philippe Lestriez, Mutlu Özcan, Yannick Josset

**Affiliations:** ^1^ Laboratoire Matériaux et Ingénierie Mécanique (MATIM), Département d’odontologie conservatrice, UFR d’odontologie, Université de Reims Champagne-Ardenne, Pôle Médecine bucco-Dentaire, CHU de Reims, Reims, France; ^2^ Laboratoire Matériaux et Ingénierie Mécanique (MATIM), Université de Reims Champagne-Ardenne, Reims, France; ^3^ Clinic for Masticatory Disorders and Dental Biomaterials, Center for Dental Medicine, University of Zurich, Zurich, Switzerland

**Keywords:** finite element, models, dental, motion capture, jaw relation record, periodontal ligament

## Abstract

**Introduction:**

This study observed the consequences of integrating mandibular kinematics in maxillary and mandibular teeth contact in a finite element analysis (FEA), and investigate the level of simplification of the dental models in FEA. The purpose of this study was to compare the results of finite element analysis obtained from simple to more complex dental models incorporating mandibular motion during loading phase.

**Methods:**

Six models were generated for this study. The simplest models consisted of only the crown of the tooth and an antagonist tooth with either the same properties or rigid body properties while the subsequent models incorporated the root of the study tooth and the surrounding bone. The most complex model involved the hyperelastic ligament and the other anatomical elements of the tooth and surrounding bone. Mandibular movement data recorded with the Modjaw® system (Modjaw-Technologie) were used to bring the teeth into contact and generate the loading in all models where the stresses exerted on tooth structures during the chewing process were evaluated.

**Results:**

von Mises stress and the shear stress obtained in all models, exceeded the ultimate compression strength of the materials, except for the model with the hyperelastic periodontal ligament. The forces applied to the tooth were extremely different depending on the addition or removal of anatomical elements despite the systematic study of the same teeth.

**Discussion:**

The inclusion of mandibular kinematics in the finite element analysis requires the modelling of a complex dental model as simplification generated an overestimation of the forces and stresses on the structures. Finite element dental models allow for the observation and prevention of restorative failures by numerical methods but misinterpretations caused by poorly designed models have clinical implications on estimating performance of dental restorations.

## 1 Introduction

Complex finite element models in dentistry tend to better represent the *in vivo* situation (1). In 2007, Magne (2) published a protocol for constructing dental models that has been used by many authors (3, 4) since then. In that study, a construction was presented by superimposing different parts of a tooth. The authors (2–4) identified the boundaries of each tooth structure from radiography (such as pulp, dentin and enamel) and generated the volume of each part. The entire dental volume was created by the superposition of the volume of each part but generally the boundaries of the pulp did not correspond to the lower limit of the dentin and the upper limit did not correspond to the lower limit of the enamel. This lack of concordance between the boundaries give rise to mesh overlaps and empty space. Consequently, these zones became stress concentration zones during the finite element calculation. The Boolean operations used at this stage of the construction made it possible to superimpose the means of two boundaries and generate only one with perfect congruence (2–4). A first approximation was thus included in the model by this means. A previous study (5) showed that it was possible to exclude this approximation and use anatomical configuration with a single dental volume including different structures such as enamel, dentine or pulp. The complexity of the maxillomandibular system does not make it easy to understand the diffusion of forces in occlusion studies (6, 7). These studies opted for a simple loading configuration of their models and applied a force in a defined direction to perform the calculations. In 2016, Dai et al. proposed a force analysis on the maxillomandibular structure following the integration of mandibular motion (8). However, it was more focused on muscle forces and did not incorporate sufficient accuracy for a finite element analysis (FEA) of tooth structures.

Currently, the literature does not present a study of a dental finite element model that has integrated the mandibular motion of the same patient to define the force applied but the Boolean approximations and oversimplified models were used in multiple studies published recently (4, 9–12).

The methods of recording mandibular movement did not have the precision necessary to make use of their data in the FEA. On the other hand, the photographic and cineradiographic methods have been abandoned due to the ionizing radiation that the patients had to undergo during the recordings. The magnetic methods (13), such as kinesiographic recordings (K7/CMS; (Myotronics, Kent, WA, USA) (14, 15) is rather used for the study of the muscles tension since chaotic recordings do not allow displacement curves to be obtained exploitable. Subsequently, the optoelectronic methods (16–18) or the ultrasound systems (8, 19–22) were described with an accuracy of 50 to 100 µm and with the problem of loss of tracking due to the unidirectional characteristics of the LED beams (23, 24). Finally, all these systems used closed softwares that do not allow the data to be extracted and used with other softwares. Today, one open software, Planmeca 4D Jaw Motion (Planmeca, Helsinki, Finland) with 4D-CBCT system, allows for data extraction. The system meshes the ultrasonographic, intra-oral scans and CBCT images to present a dynamic 3D jaw motion simulation of the patient. The only flaw is the need for CBCT acquisition which induces systematic irradiation of the patient.

The system Modjaw® (Modjaw®-Technologie, Sainte Hélène du Lac, France) invented and marketed in 2018 by Dr. Maxime Jaisson, allows for 2D or 4D occlusion analysis with recording a dynamic mapping of maxillo-mandibular contact points, occlusion curves, Bennett angles or condylar slopes. It can be used as a virtual articulator, a facebow and as an axiograph in addition to its function as a mandibular and the temporo-mandibular joint movement recorder, allowing for all motions to be observed in real time and using the data directly with dental CAD/CAM systems. It is currently the only system that makes it possible to merge 3D model, 4D motion, CBCT and facial scan with an accuracy of around 10 µm (25) and without requiring the use of x-ray for data acquisition.

Despite advances in mandibular motion measurement systems in recent years, no study has attempted to use such motion data in FEA to investigate stresses on dental structures. This limits the approximate theoretical data used in all models in the literature described above. There is in fact no longer any need to apply a force to the occlusal surface with a totally subjective value, point of impact and direction. The application of movement induces contact under force, where the direction and location of the force are identical to that found in the patient clinically. This makes it possible to start calculations with a more realistic situation. However, it is important to verify the results obtained during the application of the movement to ensure that they do not induce errors or aberrations in the results, allowing for a reliable stress analysis.

The aim of this study therefore was to observe the results of loading a dental FEA model with mandibular movement and assess the level of simplification possible for this model along with the consequences for stress analysis of dental structures. A comparison of six models, from the simplest to the most complex, was analysed in FEA. The impact of the simplification of the model integrating mandibular kinematics was also evaluated. The hypothesis was that the maximum von Mises compressive stress, maximum shear stress, maximum contact pressure and maximum force measured on each model would not differ significantly despite the complexity of the model and that the results would be consistent with the clinical situation when recording mandibular movement.

## 2 Materials and method

One patient data was used in this study. The selection was based on data from the clinical examination of a practitioner using Modjaw® v 2.0.543 (Modjaw-Technology) where several movement recordings were carried out and comparisons were made with classical techniques for locating occlusal contacts on multiple patients during the calibration phase. Patient selection was based on the absence of dental restorations on all teeth and class 1 occlusion. All patient models, acquisitions and recorded data used in this study were executed during the dental examination unrelated to this work. No intervention was performed in this study. This was a retrospective study for which informed consent was obtained from the patient in accordance with the Declaration of Helsinki for the use of these data.

### 2.1 Mandibular movement recording

**Figure 1:**
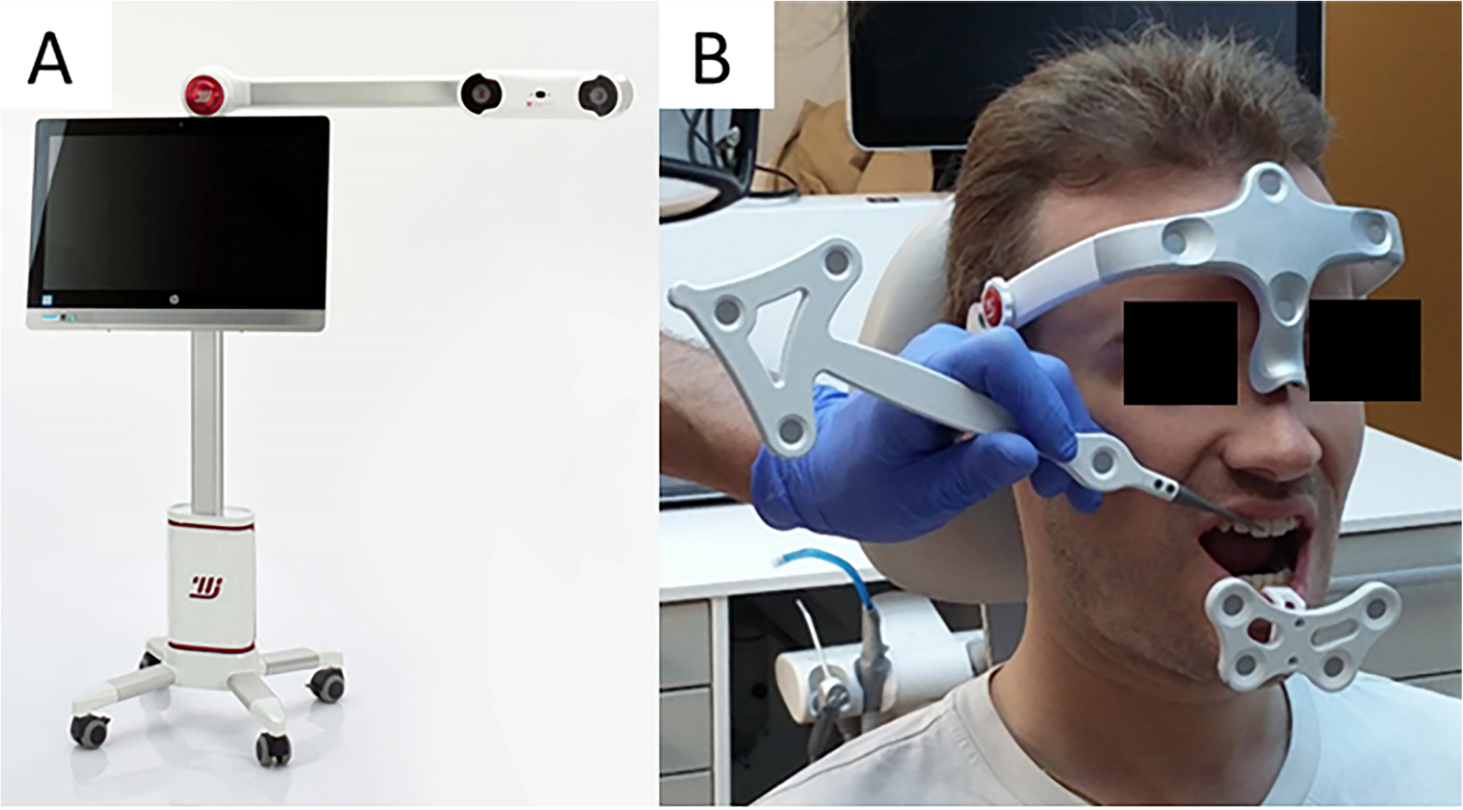
Modjaw system **(A)** camera and computer and **(B)** Set up of the headband, the mandibular gutter with reflector and taking the reference points with the stylus into consideration.

**Figure 2:**
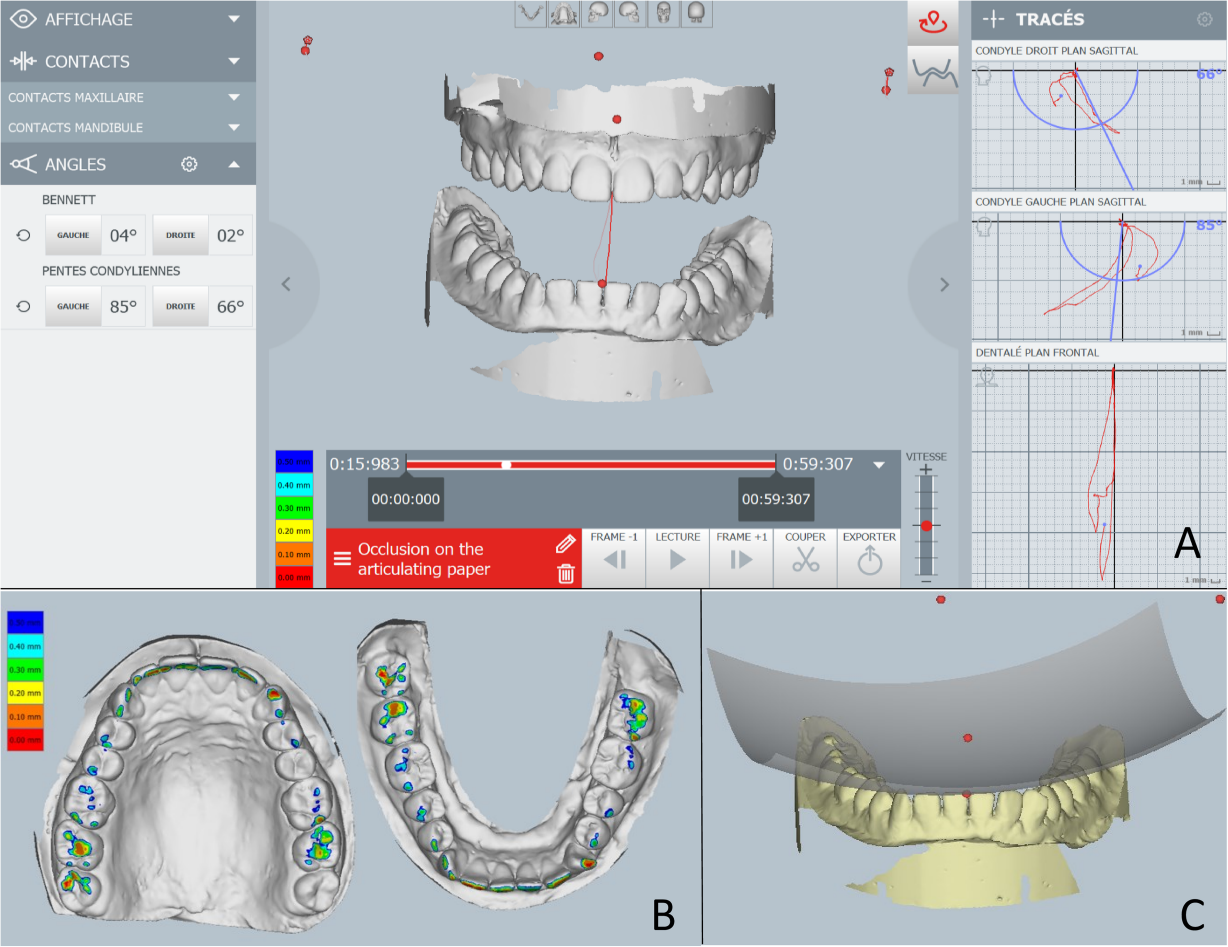
Images of the Modjaw system. **(A)** Real time motion visualization with condylar angle information on the left and the movement scheme on the right. **(B)** The occlusal contact intensity representation with different colours. **(C)** Occlusal curves illustration.

**Figure 3:**
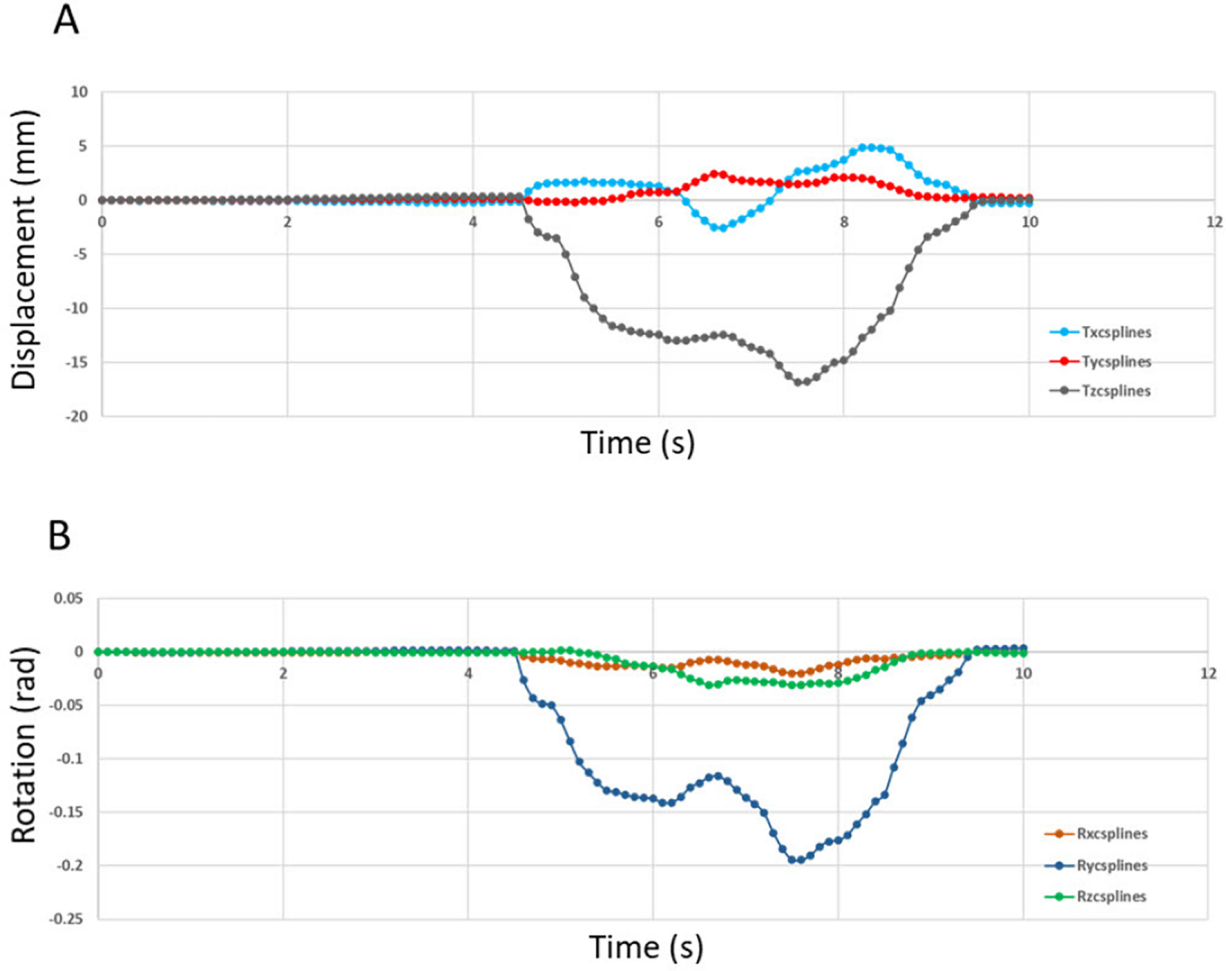
Displacement (in mm) imposed at the reference point of tooth 47 (0,0,0) in the reference frame of MODJAW®. **(A)** Motion data of the translation movement and **(B)** Motion data curve of the rotation movement.

The Modjaw® v 2.0.543 (Modjaw®-Technologie, Sainte Hélène du Lac, France) was used to record the mandibular motion. The system integrated a high-resolution camera coupled with touchscreen computer (HP EliteOne 800 G5, 23.8-inch computer with Windows 10 Professional operating system) (Figure 1A) headband and mandibular splint components with the reflector. The headband represented the fixed benchmark on the nasion where a plastic splint was held on the vestibular side of the mandibular teeth with temporary resin in order not to interfere with occlusion. A butterfly-shaped reflector fixed in front of the tray allowed for recording the movements of the mandible in relation to the position of the head. The STL files of the maxillary and mandibular cast models and the occlusion of the latter were recorded with an extra-oral scanner (series 7, Dental Wings, Strauman® Group, Montreal, Canada) and were imported into Modjaw®. The correspondence between these files and the anatomical elements on the patient was achieved using a specific stylus which made it possible to indicate the position of anatomical structures including the paracondylar points, suborbital, interincisal and subnasal point to the camera (Figure 1B). The software identified the correspondence of each point on the STL files of the plaster models. Once the system was set up, the practitioner asked the patient to perform a propulsion, deduction, and latero-deviation movement to verify the correct registration. Once it was validated, chewing movements were performed. Mandibular and condylar displacements were visualized in real time during mastication (Figure 2). For this study, the displacement during open/close moments were registered using an extra thin articulation paper as reference. The practitioner had asked the patient to reproduce this movement several times during his examination and only a part of this open/close movement with dental contact has been selected in order not to have too long calculation time. The intensity of the contact points could not be verified but identified based on the photos made by the practitioner. This step made it possible to validate the similarity of the contacts observed on the Modjaw® screen and in the patient even though this study took place without the patient. The translation and rotation data of the Modjaw® models (Figure 3) were transferred to the FEA software (Abaqus, Dassault Sytèmes® Simulia Corp®, Paris, France) after matrix calculation where the motion was applied to the mandibular tooth.

### 2.2 3D finite element model construction

#### 2.2.1 Acquisition of the anatomical data and segmentation

**Figure 4:**
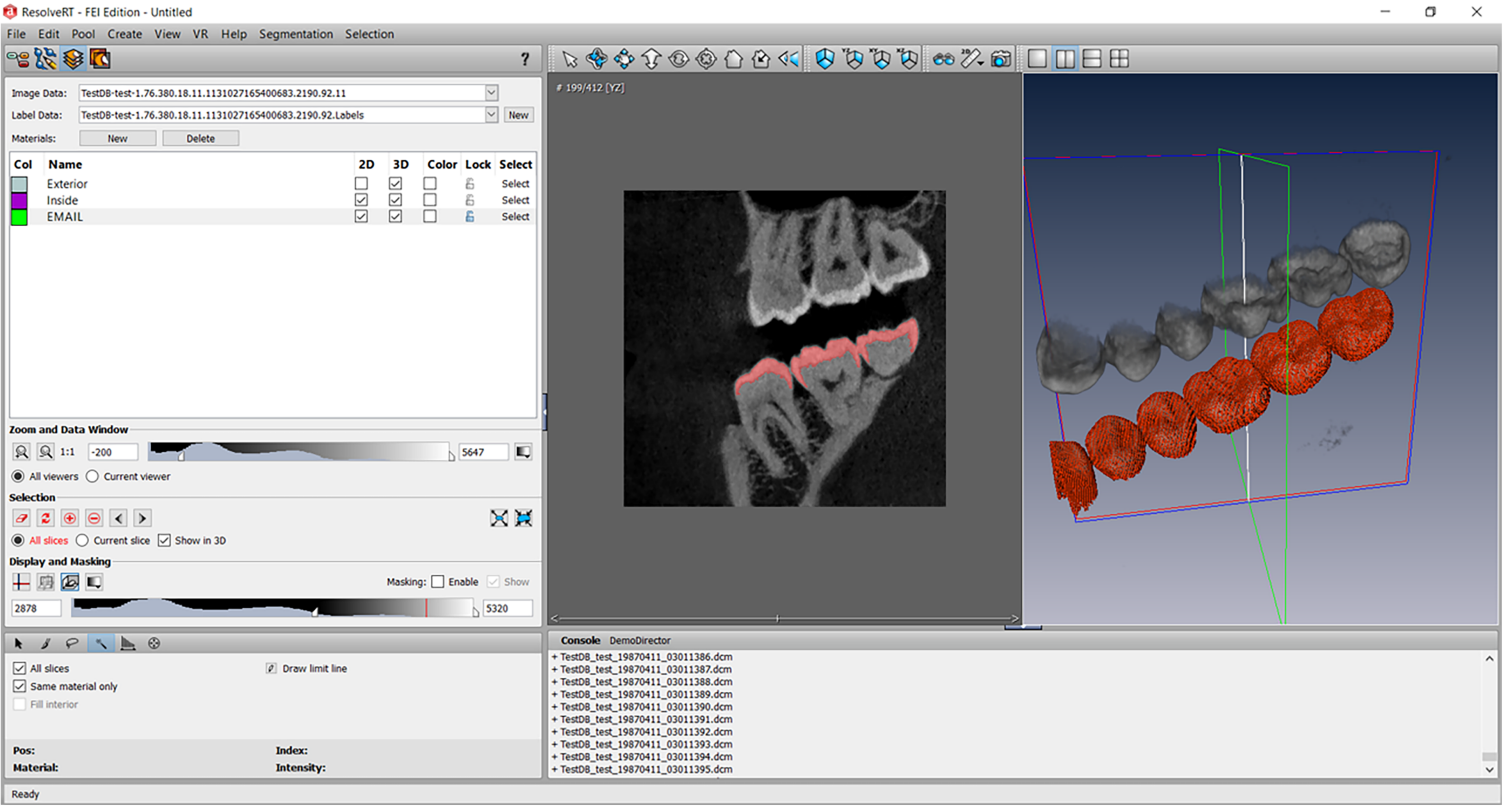
Screenshot of the Amira software segmentation of enamel on all views of the CBCT acquisition according to the gray level of the structures. Other structures were segmented using the same method.

A CBCT acquisition (NewTom® Giano, Verona, Italy) of the patient’s maxillomandibular system was used to design the 3D finite element model where 822 slices of 0.2 mm were obtained with this acquisition. The region of the maxillary and mandibular right first molars was selected. Segmentation of the entire second maxillary molar (17) and mandibular molar (47), and a small part of the surrounding bone was performed using the image processing software (Amira®, Visage Imaging GmbH, Berlin, Germany) (Figure 4). Different parts of dental anatomy (enamel, dentin, pulp) and surrounding periodontal ligament and bone were separated pixel by pixel using radiographic grey levels. Semi-automatic or manual methods were used by extraction, interpolation and progressive edge filling for the segmentation of these parts on each CBCT slice. A mask was obtained for each part: enamel, dentin, pulp, bone. The mask of periodontal ligament was built with an extraction of 0.25 mm in bone part around the root of the tooth. The Amira® files were converted in a mesh and exported (Rapidform®, Inus Technology, Seoul, Korea) for the construction of a 3D CAD model.

#### 2.2.2 Volumetric mesh and material properties

**Figure 5:**
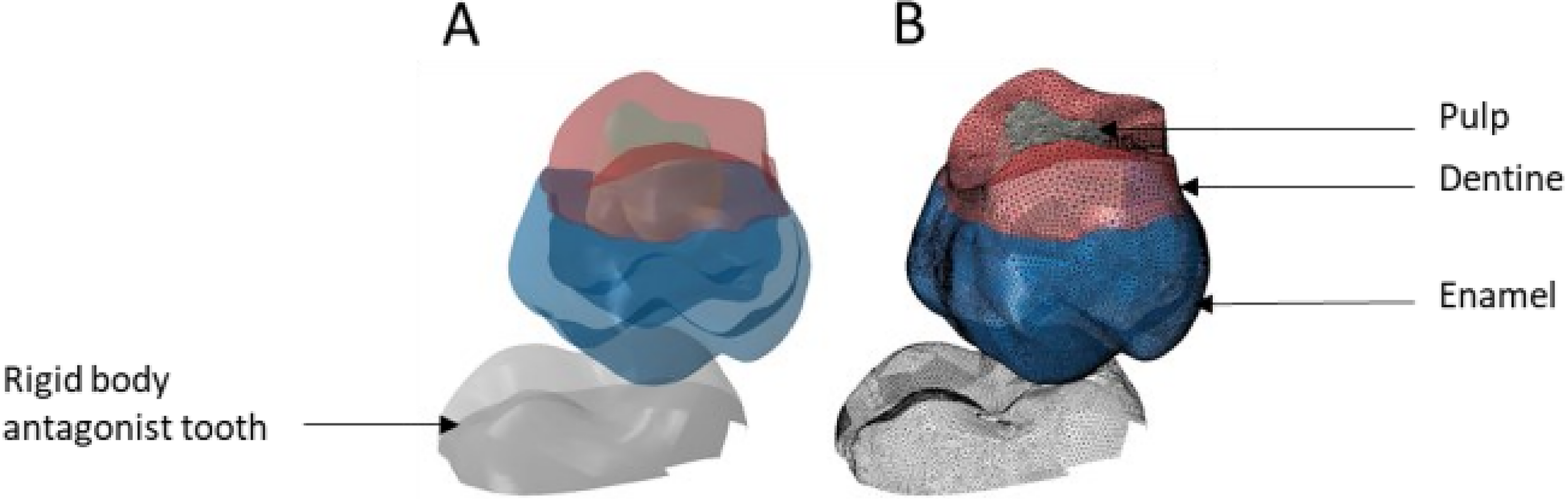
Finite element representation of the model 1 with the tooth 17 cut from the bone junction, constrained in all directions at the base of crown and considered homogeneous, linear, isotropic, and elastic. 47 was considered rigid body. **(A)** Visualisation of the different materials and **(B)** Volumetric mesh.

**Figure 6:**
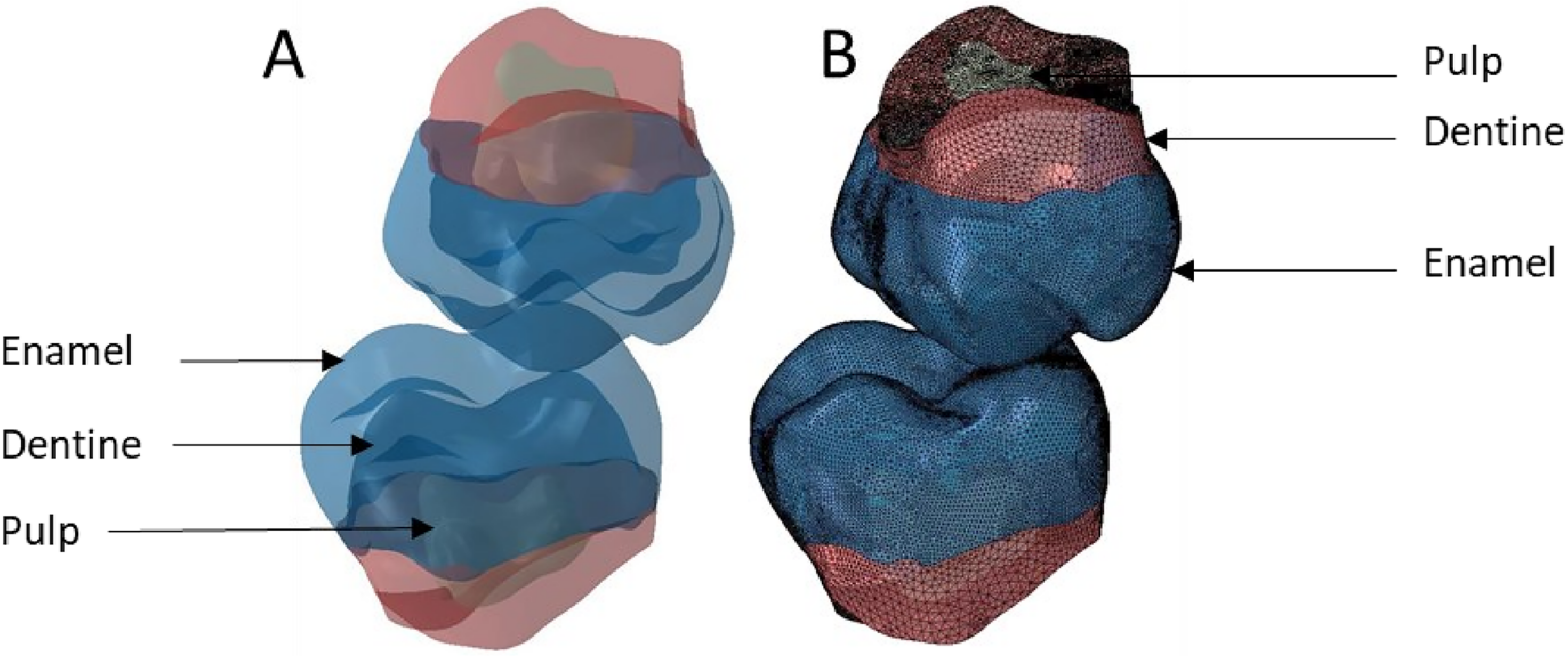
Finite element representation of the model 2 with teeth 17 and 47 cut at the bone junction and considered homogeneous, linear, isotropic, and elastic. **(A)** Visualisation of the different materials and **(B)** Volumetric mesh.

**Figure 7:**
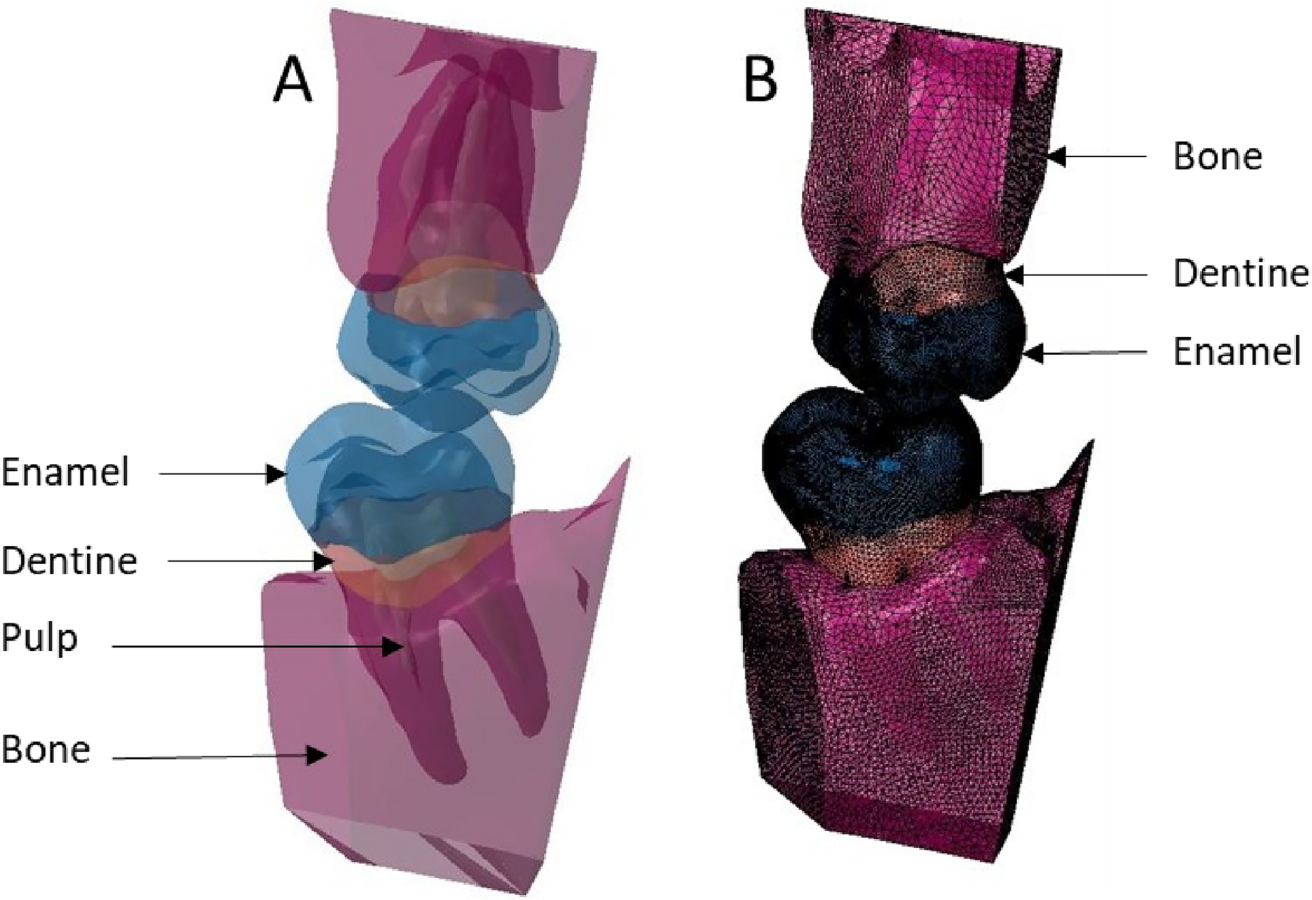
Finite element representation of the model 3 with the dental and bony structure of the 17 and 47 and without periodontal ligament. All the materials were considered homogeneous, linear, isotropic, and elastic. **(A)** Visualisation of the different materials and **(B)** Volumetric mesh.

**Figure 8:**
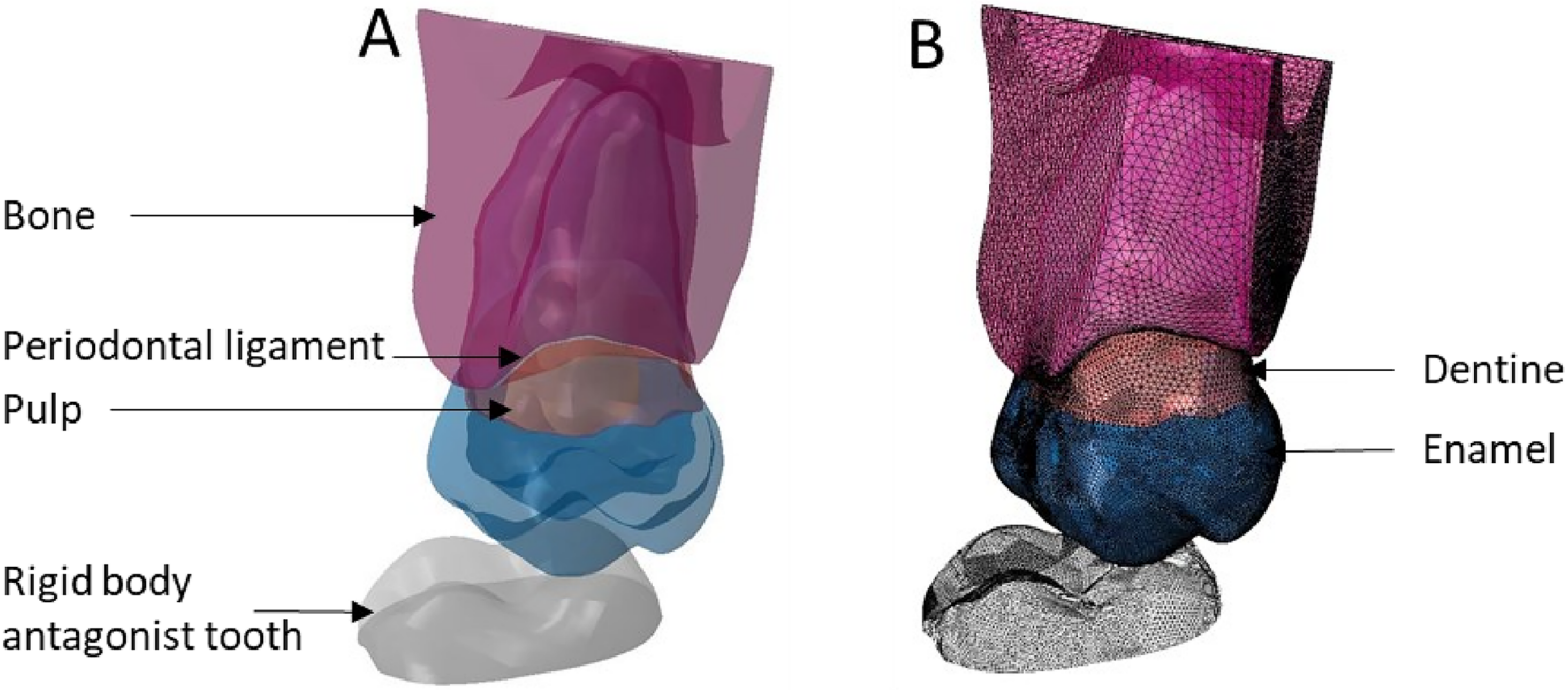
Finite element representation of the model 4 with the tooth 17 considered homogeneous, linear, isotropic, and elastic for the dental and bony structures and hyper-elastic for the periodontal ligament. The 47 was considered rigid body. **(A)** Visualisation of the different materials and **(B)** Volumetric mesh.

**Figure 9:**
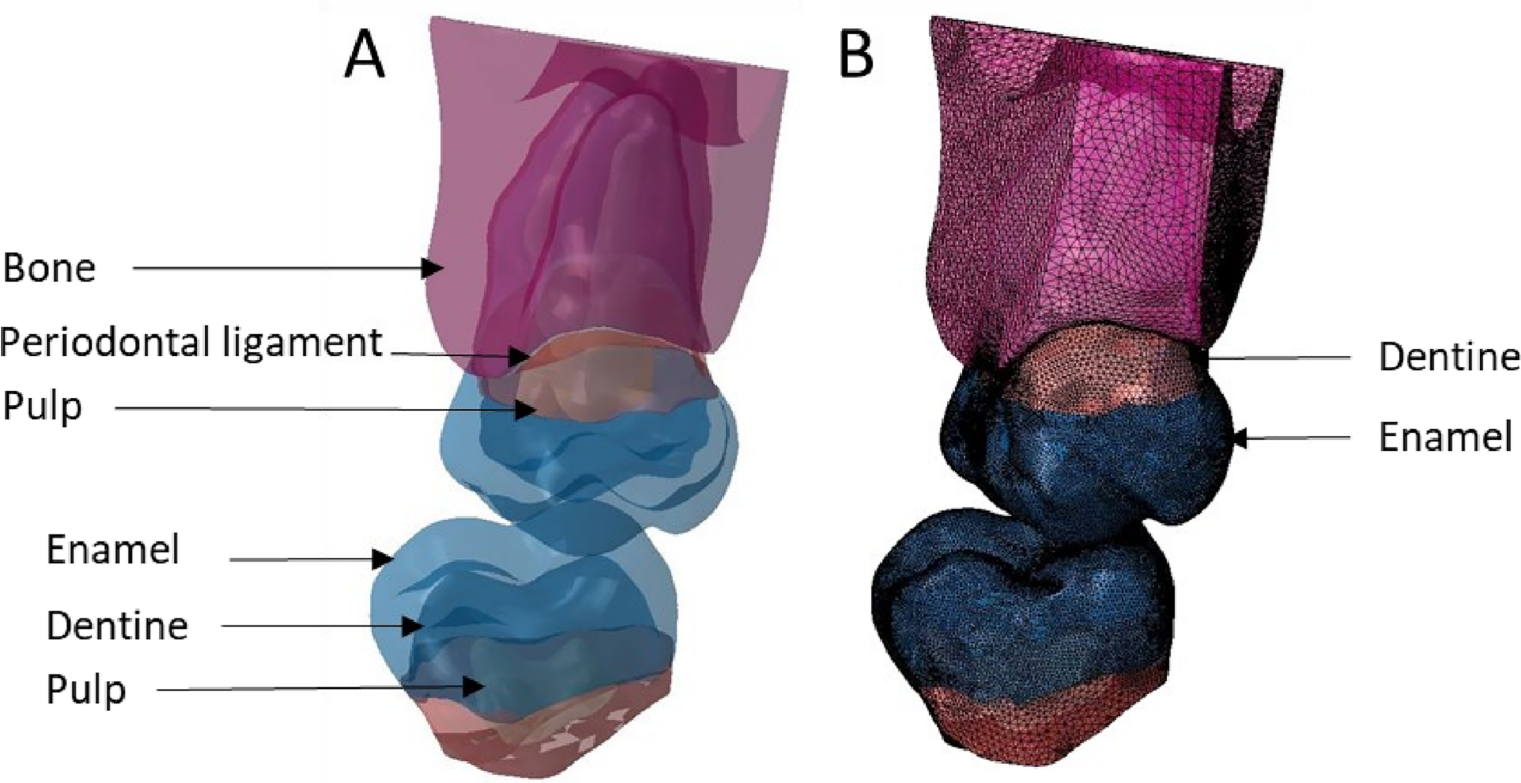
Finite element representation of the model 5 with the tooth 17 considered homogeneous, linear, isotropic, and elastic for the dental and bony structures and hyper-elastic for the periodontal ligament. For the 47 the structures were cutting from bone junction and the materials were considered homogeneous, linear, isotropic, and elastic. **(A)** Visualisation of the different materials and **(B)** Volumetric mesh.

**Figure 10:**
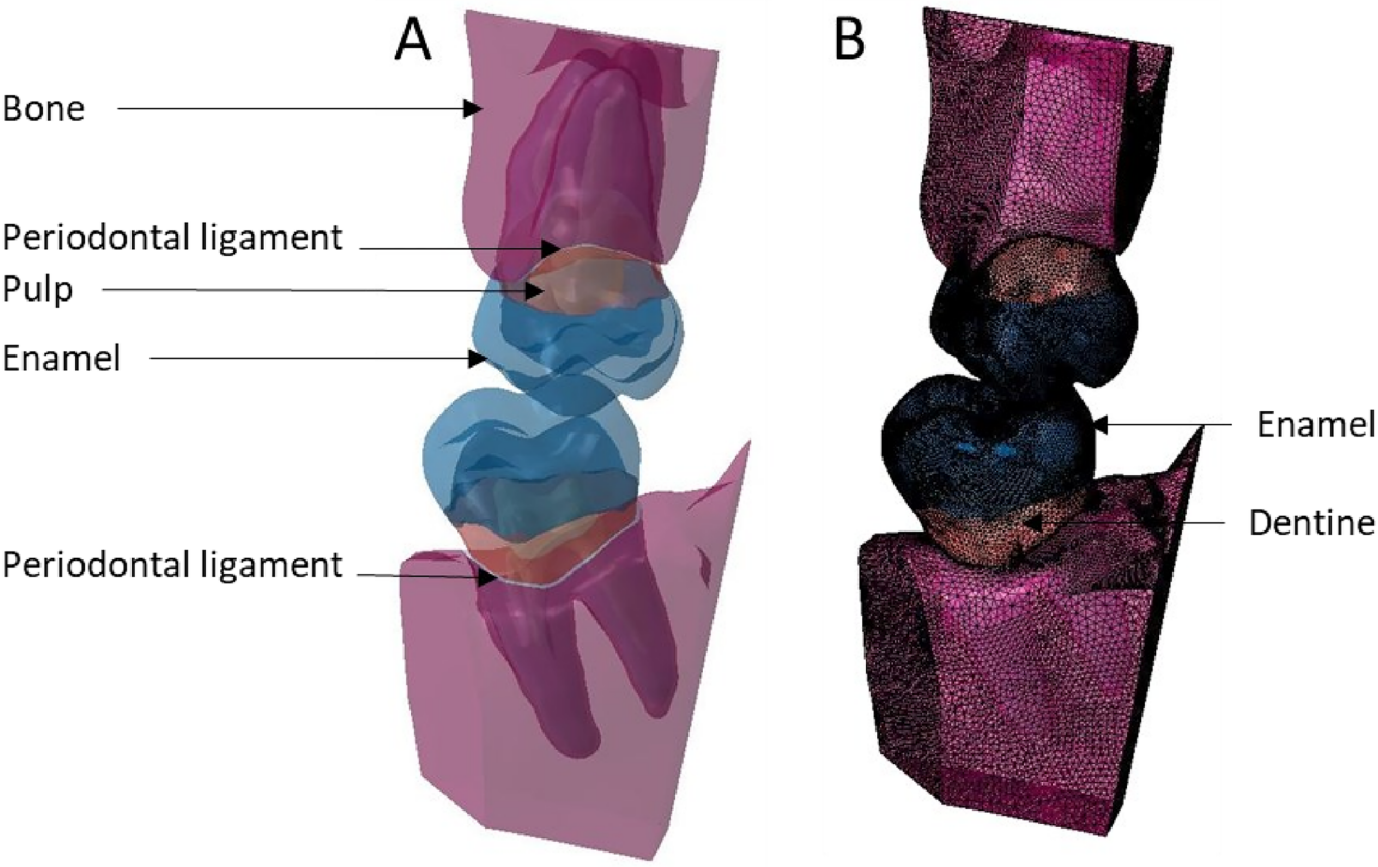
Finite element representation of the model 6 with the teeth 17 and 47 considered homogeneous, linear, isotropic, and elastic for the dental and bony structures, and hyper-elastic for the periodontal ligament. **(A)** Visualisation of the different materials and **(B)** Volumetric mesh.

The model was constructed according to the previous study (5). The first step taken with Rapidform® was to restore congruency between the masks (pulp-dentin-enamel). The junctions between enamel and dentin and between pulp and dentin were represented by the 3D surfaces created. A closed solid was then created after thorough cleaning. Dental models found in the literature are made up of superimposed of pulp, dentin and enamel volumes (2, 26, 27). In this study, for each tooth, a single volume was divided into several parts with all included structures (enamel, dentin, pulp, periodontal ligament, and bone). The surface of masks represented the joints between these materials in the same volume. This method of construction with the distribution of different materials of the same volume avoided the stresses at the interface joint and removed the need to calculate approximation with Boolean operations as in other methods described previously (2). Model was imported into Abaqus® (Dassault Systèmes® Simulia Corp®, Paris, France) after volumetric mesh creation. Mesh optimisation was performed, and a size of 0.025 mm was chosen. Six situations were tested (Figures 5–10) where in each model, the mesh size and density of each anatomical part were identical.

Starting from model 1 with only the crown part and the rigid body antagonist tooth, the anatomical elements of the root and the periodontal environment were added along with the properties of structures that were modified from rigid to elastic or hyperelastic up to model 6. From the simplified model to the complex model, different anatomical parts were added in the model increasing the number of elements but not changing their size or density. C3D4 elements represented tetrahedral deformable elements and these were the same elements with hyperelastic properties while R3D3 elements were triangular rigid surface (Tables 1, 2).

**Table 1:** Model conception information.

Model	Tooth	Cutting plan	Materials	Materials properties	Finite element
1	17	Bone junction	Enamel	Homogeneous, linear, isotropic and elastic	857,401 linear tetrahedral elements of type C3D4
			Dentin		
			Pulp		
	47	Occlusal part only	None	Rigid body	13,694 linear triangular elements of type R3D3
2	17	Bone junction	Enamel	Homogeneous, linear, isotropic and elastic	16,76,472 linear tetrahedral elements of type C3D4
	47		Dentin		
			Pulp		
3	17	None	Enamel	Homogeneous, linear, isotropic and elastic	29,08,338 linear tetrahedral elements of type C3D4
	47		Dentin		
			Pulp		
			Bone		
4	17	None	Enamel	Homogeneous, linear, isotropic and elastic	220,231 linear tetrahedral elements of type C3D4H hybrids for the hyperelastic periodontal ligament, with 18,87,761 linear tetrahedral elements of type C3D4
			Dentin		
			Pulp		
			Bone	Hyperelastic (PDL)	
			Periodontal ligament		
	47	Occlusal part only	None	Rigid body	13,694 linear triangular elements of type R3D3
5	17	None	Enamel	Homogeneous, linear, isotropic and elastic	220,231 linear tetrahedral elements of type C3D4H hybrids for the hyperelastic periodontal ligament
			Dentin		
			Pulp		
			Bone	Hyperelastic (PDL)	
			Periodontal ligament		
	47	Bone junction	Enamel	Homogeneous, linear, isotropic and elastic	706,677 linear tetrahedral elements of type C3D4
			Dentin		
			Pulp		
6	17	None	Enamel	Homogeneous, linear, isotropic and elastic	406,208 linear tetrahedral elements of type C3D4H hybrids for the periodontal ligaments of both teeth, and 38,47,987 linear tetrahedral elements of type C3D4
	47		Dentin		
			Pulp		
			Bone	Hyperelastic (PDL)	
			Periodontal ligament		

**Table 2:** Materials properties used in the finite element analysis.

Material	Elastic modulus (MPa)	Poisson’s ratio	Elastic limit (MPa)				References
Enamel	80000	0.33	384				(10, 28)
Dentin	15000	0.31	297				(10, 29)
Pulp	2	0.45	294				(30)
Bone	13700	0.3	167				(28)
Periodontal ligament (hyper elastic)	0.23	0.49	**Prony constant**				(31)
			Reduced time τ (s)	0.0025	0.1	0.5	
			Bulk modulus K	0.155	0.4	0.15	
			Shear modulus G	0	0	0	

#### 2.2.3 Boundary conditions and finite element analysis

**Figure 11:**
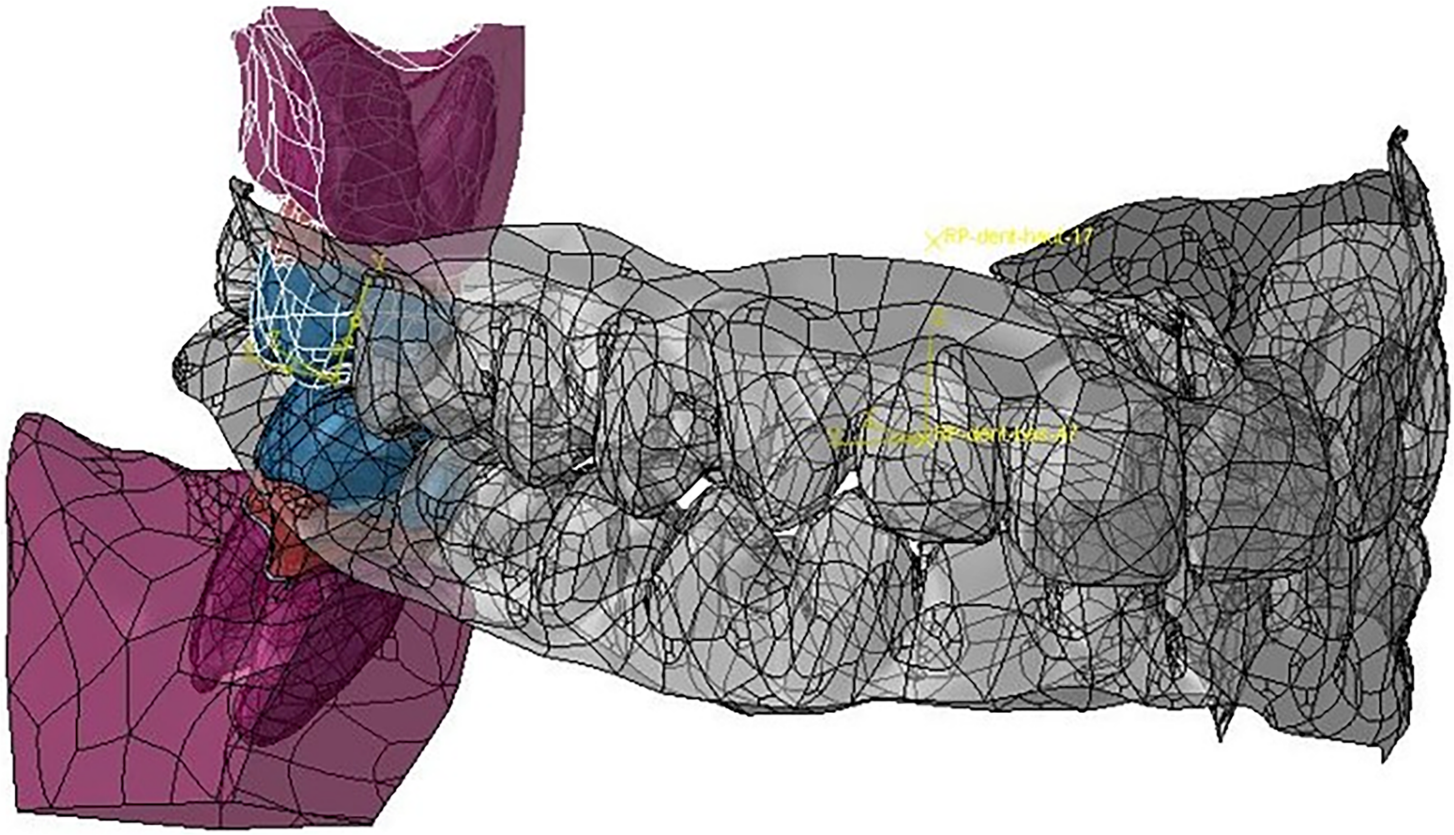
Matching of the Modjaw maxillo-mandibular model and the model built for the finite element analyses of teeth 17 and 47.

The boundary conditions for models 3 through 6 were the same with all nodes on the upper bone surface of tooth 17 constrained in all directions. In all these configurations, the coefficient of friction between the teeth was 0.1 (32, 33). Thereafter, Modjaw model and the constructed model were superimposed, and the same reference point was used for the motion data (Figure 11). For each tooth of the model, different movements of the jaw were transcribed by matrix transformations of rigid bodies making it possible to obtain quaternions applied in the FEA. From these matrix results, other transformation and resolution routines were set to obtain the displacement and rotation vectors applicable in the Abaqus® software. These vectors were applied to tooth 47 in order to induce a movement. The motion of 47 created a contact between the teeth and generated the load on the tooth 17. The loading intensity and location was defined by the motion step and represented the patient`s occlusion. The load direction was defined by motion direction and the contact surface between the teeth. No modification was performed as the aim was to have a realistic situation. FEA was used to evaluate the von Mises stress, the elastic strain, and the displacement of the model structures. No force was applied to the teeth during analysis and the data collected were the results of tooth contact induced by mandibular movement.

## 3 Results

**Figure 12:**
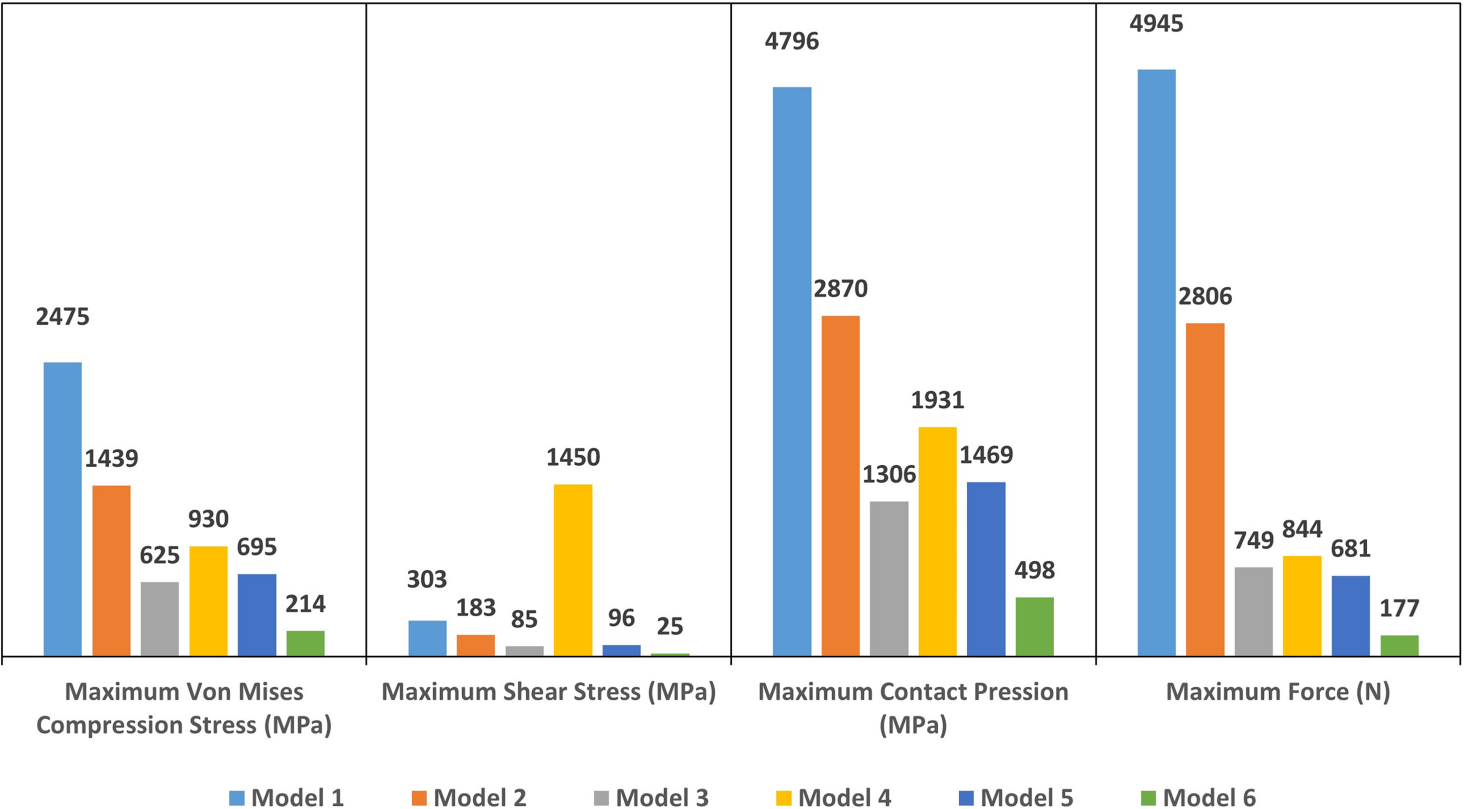
The maximum Von Mises stress (in MPa), the maximum shear stress (in MPa), the maximum contact pressure (in MPa) and the maximum force (in N) measured on enamel in each model.

**Figure 13:**
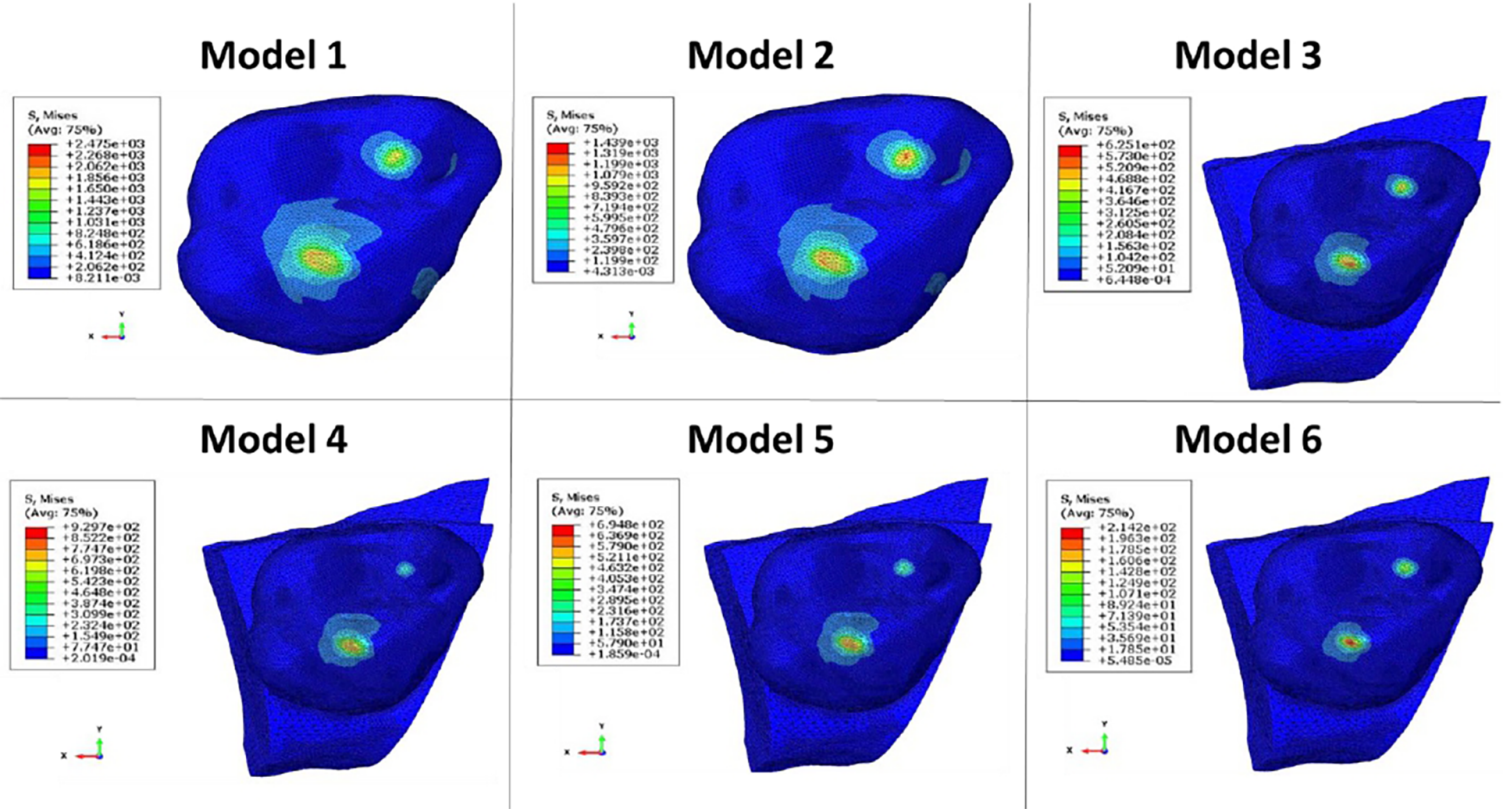
Von Mises stresses measured on the enamel during mandibular movement in different models studied.

**Figure 14:**
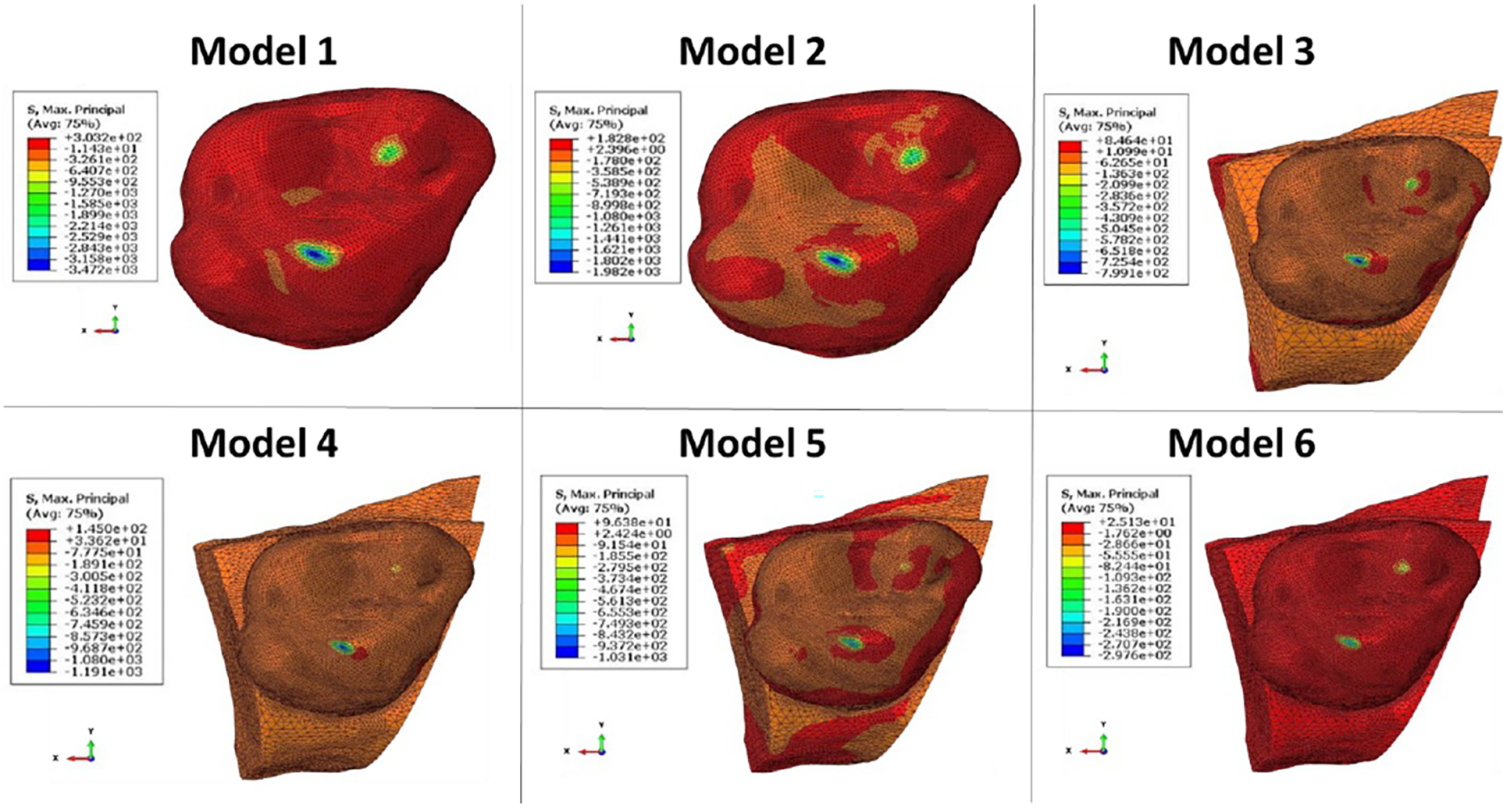
The maximum shear stresses measured on enamel during mandibular movement in different models studied.

The computation time was 32,852 cpus for the first model and reached 8,24 E^+05^ cpus for the 6th model. Figure 12 shows the maximum von Mises stress, maximum shear stress, maximum contact pressure and maximum force measured in each model. These were all observed in the enamel as the highest values appeared in enamel. Figures 13, 14 show the von Mises stresses and the maximum shear stresses measured on the enamel during mandibular motion for the different models, respectively. The force was approximately 5,000 N for model 1, around 3,000 N for model 2 and exceeded 600 N for models 3, 4 and 5. For model 6, the maximum resulting force was 177 N. The von Mises stresses observed in models 1 to 5 greatly exceeded the elastic limit values of enamel in compression (384 MPa) (10).

## 4 Discussion

In this study, tooth displacement defined the load on the dental structure. The contact point locations have been defined by the direction of mandibular movement in the selected patient in in Class I occlusion and represented the patient’s actual occlusal position. The maximum von Mises stress, maximum shear stress, maximum contact pressure and maximum force were measured in the maximum intercuspation position. In the literature, mandibular kinematics has not been used directly in order to determine the stress on tooth structure. In previous studies, occlusion force (20 to 1,000 N) was measured during maximum intercuspation time and applied vertically or obliquely to the surface of the dental model (4, 6, 7, 9). However, the measurement of the occlusal force is controversial as the standard way to measure this force is with piezoelectric transducers or strain gauges. These systems however, prevent the patient from having a perfect occlusion. They create an extra thickness on the tooth through which the forces are measured and generate a controlled rather than physiological occlusion. The values of the occlusal force described in the literature varies greatly ranging from 80 (34) to 1,400 N for patients with bruxism (35, 36) but do not reflect the situation of the patient for whom the finite element model is designed. Some systems such as the T-Scan (37) (T-Scan III, Tekscan Corp, Boston, Mass., USA) only give a relative value in relation to the patient’s entire arch. The most important contact zones can be observed with the chronology of their appearance, but the value indicated is not an absolute value as in the case of measurement with one of the piezoelectric transducers or strain gauges. In this study, mandibular kinematics were measured during occlusion on the extra thin articulation paper. The required occlusal force was not too high in the tested situation. The forces greater than 1,000 N occurred only during 100% voluntary occlusion and in the molar regions (35), but overall they were always below this value. This proves that models 1 and 2 cannot be exploited since the forces were well above 2,000 N.

The motion brings the teeth into contact in a continuous motion while the teeth in contact generates a force applied to the structures. The force applied exactly on the occlusal points during the clinical examination involves the action of the muscles as it is this action that allows to direct the mandible of the patient. The achieved model largely improved the quality compared to the models found in the literature (26, 27). Direct use of mandibular kinematic data requires high degree of accuracy in the modelling and the recording the mandibular motion. Without this accuracy, the integration of these data into the FEA generated excessive overlaps of the mesh of the two opposing teeth that resulted in computational errors.

In this study, different mesh configurations were tried to obtain a sufficiently accurate model in order to complete the calculation without abortion. From model 1 to 6, the complexity and validity of the construction increased. In models 1 and 4, tooth 47 was considered as a rigid body and all displacements were supported by tooth 17 which also supported stresses beyond the elastic properties of the materials. Since enamel is a brittle material, its Young’s modulus was defined with a variation between 70,000 and 13,300 MPa depending on the composition of its crystalline fraction (38, 39). These properties change with the distance to the pulp and the direction of the hydroxyapatite crystals (40). The ultimate compressive strength of enamel has been described to be between 100 and 400 MPa with a low deformation capacity of ∼1% (39). The Young’s modulus of dentin on the other hand has been described to range between 11,000 and 40,000 MPa (39, 41) with ultimate compressive strength ranging between 250 and 350 MPa with a strain before fracture at about 2% (39). However, in the finite element models from 1 through 5 used in this study, the observed von Mises stress on the enamel was higher than the ultimate compressive strength of the structures. Only in model 6, the results yielded a von Mises stress (214 MPa) lower than the elastic properties of the structures. Model 6 was in fact the more representative one as the bone structure and periodontal ligament were constructed for both teeth. The contact of the teeth during closing, generated a displacement of each tooth in its alveolar socket with deformation in the periodontal ligament. These displacements reduced the load on the structure of each tooth. The variation in periodontal ligament property values found in the studies demonstrates the difficulties of understanding the stress distribution in this structure *in vivo* (42). The difference between models 3 and 6 indicated the importance of modelling of the periodontal ligament in 3D dental models. The exclusion of hyperelastic elements generated forces 4 times higher and the stresses supported by the enamel were once again greater than its elastic limit. As with the results previously described (43, 44), the omission of the construction of the periodontal ligament because of its thin and weak elastic properties, was due to the fact that it was not the main objective of this research. Furthermore, simplification of the models would induce erroneous results on stress distribution. The comparison of models 4, 5 and 6 however showed the importance of constructing the integral anatomy of the opposing tooth. The construction in section and the exclusion of the calculation with the rigid body representation gave the same result as for model 3. The constraints were supported only by the tooth studied and generated an overestimation of the values. In this regard, the present study complements previous observations (43, 44) and highlights the use of a model loaded with mandibular kinematics even though these are represented with isotropic properties and with a simplified geometry. It is obvious that the FEA are only a representation of reality and cannot for the moment, for reasons of calculation time and difficulties of understanding, have the accuracy of a real anatomical situation (43).

The stress distribution changes with the loading conditions. Loading with a point force or a rigid sphere can, as discretization properties, concentrate the stress on the loading point and give unrealistic stress values (45). To avoid misinterpretation, it is important to consider the area of the loading surface. The same loading force applied on a small area will not give the same stress results compared to an application to a larger area (45). Conventional loading by applying force to a point or a surface of the model cannot represent a real state because currently there is no means of measuring the occlusal force to give a quantitative value of this force. The tools described in the literature only bring a beginning of information because they are relative forces observed among all the forces measured on a dental arch. Moreover, it should be noted that in order to measure the occlusal force, the literature suggests measuring instruments (e.g., strain gauges) be placed between the teeth (36, 46). However, this does not allow the measurement of a physiological state of occlusion. With the Modjaw system, the splint bonded to the mandibular teeth is located on the vestibular surface of the teeth and therefore does not interfere with the patient’s occlusion at all. The patient can clench his or her teeth or chew food as usual throughout the recording of the movement.

The precision of Modjaw® cameras on intermaxillary relations was 9.7 ± 1.76 µm, and the trueness was 10.8 ± 1.40 µm (25). The accuracy of the camera and software calculations also allowed for the mandibular motion data to be used in the FEA. Simultaneous recording of head movement and the mandibular movement was also a key element in calculating the real motion of the mandible. In various systems described in the literature (47), the markers were placed on the skin on the chin, forehead or temples. Even if the patient is warned about the importance of not having facial expressions, it is difficult to control the skin movements during chewing movements. The Modjaw® system consists of a headband with a nasal support. The correct support behind the head and minimal support on the nose prevents excessive movement of the band. This reduces noise during recording and increases the accuracy of the recording of mandible movement. The patient’s proprioception interferes with the strength and position of the occlusion and can provide more information for the finite element dental model. Applying the theoretical mandibular trajectory to a dental model constructed from a skeletal micro-CT acquisition may overestimate the resulting force and occlusal stresses of the specimen (48). Therefore, additional work is needed with the patient’s own movement to consider dental anatomy, wear in veneer morphology and variations in tooth position and pressure over time. The different configurations of the dental models used in this study showed the importance of including as many anatomical parts as possible. During mandibular motion and maxillo-mandibular occlusion, several deformations take place during the bite process and if the models do not consider this information, the results of the forces exerted upon the dental structures are overestimated. Without a construction of the overall anatomic parts of the tooth, the periodontal ligament with hyperelastic properties or the bony structures, the forces exceeded the elastic limits of all individual structures. It is therefore not possible for brittle structures such as enamel and dentin to be subject to such high forces while their deformation rate is very low.

Understanding the importance of modelling the anatomical structures when using mandibular kinematics to feed the finite element model also allowed us to demonstrate that the results obtained in the literature may overestimate the real situation. Maximal voluntary bite force assessment has been investigated previously (45, 49, 50) but the use of this information in the model with a simple dental crown without modelling of other anatomical elements or with an antagonist tooth having rigid properties does not allow for an accurate assessment of stresses on structures and complicates the comparison between studies.

This work has enabled us to verify the need to include all anatomical elements in the dental model when using mandibular kinematics. Further studies should be carried out to determine the impact of structural properties, repeatability of the protocol with several patients and also compare the results with an experimental model. The process of determining the accuracy of a model is a representation of the real world and depends on the intended use of the model (51). Dental models are often intended to understand the reasons and processes of failure of either the tooth structure or direct/indirect restorations. As such, overestimating forces with overly simplified models will not be an accurate reflection of the real situation. Finally, an even more complex model, considering the deformation of the mandibular arch, should take place in order to better understand the stress dispersion during mastication.

## 5 Conclusion

Based on the findings of this study, the integration of mandibular kinematics into the dental finite element analysis required the use of all anatomical structures (enamel, dentin, pulp, periodontal ligament and bone) in the finite element model construction. The omission of some of these structures during the construction of the model for the sake of simplification overestimates the forces experienced on the dental structures. This is an essential point that also calls into question the results of biomechanical studies on dental restorations and implants in the literature.

## Data Availability

The original contributions presented in the study are included in the article/Supplementary Material, further inquiries can be directed to the corresponding author.

## References

[1] Moutinho MBF Belinha J Jorge RMN. Using the Finite Element Method and Radial Point Interpolation Method to Analyze the jaw Bone (2019). p. 1–4

[2] Magne P. Efficient 3D finite element analysis of dental restorative procedures using micro-CT data. Dent Mater. (2007) 23(5):539–48. 10.1016/j.dental.2006.03.013 16730058

[3] Hussein LA. Three-dimensional-finite-element-analysis-of-different-composite-resin-MOD-inlays.pdf. J Am Sci. (2013) 9(8):422–8. 10.7537/marsjas090813.45

[4] Rodrigues MdP Soares PBF Gomes MAB Pereira RA Tantbirojn D Versluis A Direct resin composite restoration of endodontically-treated permanent molars in adolescents: bite force and patient-specific finite element analysis. J Appl Oral Sci. (2020) 28:e20190544. 10.1590/1678-7757-2019-0544 32348440 PMC7185981

[5] Özcan C Josset Y Muraille C Lestriez P Taiar R. A three-dimensional (3D) finite element model of restored molar teeth and combination of restorative material. Ser Biomech. (2017) 31(4):12–8.

[6] Borges Radaelli MT Idogava HT Spazzin AO Noritomi PY Boscato N. Parafunctional loading and occlusal device on stress distribution around implants: a 3D finite element analysis. J Prosthet Dent. (2018) 120(4):565–72. 10.1016/j.prosdent.2017.12.023 29724560

[7] Türker N Büyükkaplan US Sadowsky SJ Özarslan MM. Finite element stress analysis of applied forces to implants and supporting tissues using the « all-on-four » concept with different occlusal schemes. J Prosthodont. (2019) 28(2):185–94. 10.1111/jopr.13004 30515911

[8] Dai F Wang L Chen G Chen S Xu T. Three-dimensional modeling of an individualized functional masticatory system and bite force analysis with an orthodontic bite plate. Int J Comput Assist Radiol Surg. (2016) 11(2):217–29. 10.1007/s11548-015-1248-4 26108294

[9] Dave MM Kothari KD. Finite element analysis of patient-specific maxillary molar crown. Int J Innovative Technol Explor Eng. (2019) 8(11):2474–8. 10.35940/ijitee.K1720.0981119

[10] Dejak B Młotkowski A. A comparison of stresses in molar teeth restored with inlays and direct restorations, including polymerization shrinkage of composite resin and tooth loading during mastication. Dent Mater. (2015) 31(3):e77–87. 10.1016/j.dental.2014.11.016 25544104

[11] Dikova T Vasilev T Hristova V Panov V. Finite element analysis in setting of fillings of V-shaped tooth defects made with glass-ionomer cement and flowable composite. Processes. (2020) 8(3):363. 10.3390/pr8030363

[12] Franco ABG Franco AG de Carvalho GAP Ramos EV Amorim JCF de Martim AS. Influence of conservative endodontic access and the osteoporotic bone on the restoration material adhesive behavior through finite element analysis. J Mater Sci: Mater Med. (2020) 31(4):39. 10.1007/s10856-020-06377-7 32279130

[13] Nagpal A Abrol S Duvedi K Ruthwal Y Kashyap P. Mandibular movements: record and analysis from time immemorial. Ann ProsthodRestor Dent. (2017) 3:57–9. 10.18231/2455-8486.2017.0013

[14] Gawriołek K Gawriołek M Komosa M Piotrowski PR Azer SS. Kinematic modeling of normal voluntary mandibular opening and closing velocity–initial study. J Prosthodont. (2015) 24:279–86. 10.1111/jopr.12212 25219889

[15] Monaco A Sgolastra F Pietropaoli D Giannoni M Cattaneo R. Comparison between sensory and motor transcutaneous electrical nervous stimulation on electromyographic and kinesiographic activity of patients with temporomandibular disorder: a controlled clinical trial. BMC Musculoskelet Disord. (2013) 14:168. 10.1186/1471-2474-14-168 23672400 PMC3660267

[16] Chen CC Chen YJ Chen SC Lin HS Lu TW. Evaluation of soft-tissue artifacts when using anatomical and technical markers to measure mandibular motion. J Dent Sci. (2011) 6(2):95–101. 10.1016/j.jds.2011.03.010

[17] Raabe D Harrison A Alemzadeh K Ireland A Sandy J. Capturing Motions and Forces of the Human Masticatory System to Replicate Chewing and to Perform Dental Wear Experiments. Bristol, United Kingdom: IEEE; (2011). p. 1–6. http://ieeexplore.ieee.org/document/5999149/

[18] Xu WL Bronlund JE Potgieter J Foster KD Röhrle O Pullan AJ Review of the human masticatory system and masticatory robotics. Mech Mach Theory. (2008) 43(11):1353–75. 10.1016/j.mechmachtheory.2008.06.003

[19] Enciso R Memon A Mah J. Three-dimensional visualization of the craniofacial patient: volume segmentation, data integration and animation. Orthod Craniofac Res. (2003) 6(Suppl 1):66–71. 10.1034/j.1600-0544.2003.237.x 14606537

[20] Goiato MC Zuim PRJ Moreno A dos Santos DM da Silva EVF de Caxias FP, Does pain in the masseter and anterior temporal muscles influence maximal bite force? Arch Oral Biol. 2017;83:1-6. 10.1016/j.archoralbio.2017.06.029 28688272

[21] Kijak E Lietz-Kijak D Frączak B Śliwiński Z Margielewicz J. Assessment of the TMJ dysfunction using the computerized facebow analysis of selected parameters. Biomed Res Int. (2015) 2015:1–9. 10.1155/2015/508069 PMC4442268 26078951

[22] He S Kau C Liao L Kinderknecht K Ow A Saleh T. The use of a dynamic real-time jaw tracking device and cone beam computed tomography simulation. Ann Maxillofac Surg. (2016) 6(1):113. 10.4103/2231-0746.186142 27563619 PMC4979326

[23] Kordaß B Behrendt C Ruge S. Computerized occlusal analysis–innovative approaches for a practice-oriented procedure. Int J Comput Dent. (2020) 23(4):363–75. PMID: . 33491932

[24] Jakubowska S Szerszeń M Kostrzewa-Janicka J. Jaw motion tracking systems—literature review. Prosthodontics. (2023) 73(1):18–28. 10.5114/ps/162663

[25] Nagy Z Mikolicz A Vag J. *In vitro* accuracy of a novel jaw-tracking technology. J Dent. (2023) 138:104730. 10.1016/j.jdent.2023.104730 37777084

[26] Babaei B Shouha P Birman V Farrar P Prentice L Prusty G. The effect of dental restoration geometry and material properties on biomechanical behaviour of a treated molar tooth: a 3D finite element analysis. J Mech Behav Biomed Mater. (2022) 125:104892. 10.1016/j.jmbbm.2021.104892 34688146

[27] Mustaffa M Marghoub A Shahrbaf S Martin N. Evaluation of the stress pattern in the resin-based composite restoration of an endodontically treated premolar tooth: a finite element analysis study. J Int Dental Med Res. (2022) 15(1):53–60.

[28] Monteiro JB Dal Piva AdO Tribst JPM Borges ALS Tango RN. The effect of resection angle on stress distribution after root-end surgery. Iran Endod J. (2018) 13(2):188–94. 10.22037/iej.v13i2.19089 29707013 PMC5911292

[29] Rees JS Jacobsen PH. The elastic moduli of enamel and dentine. Clinic Mater. (1993) 14(1):35–9. 10.1016/0267-6605(93)90045-9

[30] Dejak B Mlotkowski A. Three-dimensional finite element analysis of strength and adhesion of composite resin versus ceramic inlays in molars. J Prosthet Dent. (2008) 99(2):131–40. 10.1016/S0022-3913(08)60029-3 18262014

[31] Su MZ Chang HH Chiang YC Cheng JH Fuh LJ Wang CY Modeling viscoelastic behavior of periodontal ligament with nonlinear finite element analysis. J Dent Sci. (2013) 8(2):121–8. 10.1016/j.jds.2013.01.001

[32] Ang KY Lucas PW Tan HTW. Incisal orientation and biting efficiency. J Hum Evol. (2006) 50(6):663–72. 10.1016/j.jhevol.2006.01.003 16530808

[33] Douglas WH Sakaguchi RL DeLong R. Frictional effects between natural teeth in an artificial mouth. Dent Mater. (1985) 1(3):115–9. 10.1016/S0109-5641(85)80040-3 3861435

[34] Abe I Milczewski MS Souza MA Kalinowski HJ Machuca OF Marin GC The force magnitude of a human bite measured at the molar intercuspidation using fiber Bragg gratings. J Microw Optoelectron Electromagn Appl. (2017) 16(2):434–44. 10.1590/2179-10742017v16i2808

[35] Hidaka O Iwasaki M Saito M Morimoto T. Influence of clenching intensity on bite force balance, occlusal contact area, and average bite pressure. J Dent Res. (1999) 78(7):1336–44. 10.1177/00220345990780070801 10403461

[36] Jansen van Vuuren L Broadbent JM Duncan WJ Waddell JN. Maximum voluntary bite force, occlusal contact points and associated stresses on posterior teeth. J R Soc N Z. (2020) 50(1):132–43. 10.1080/03036758.2019.1691612

[37] Aishwarya N Nagarathna C Poovani S Thumati P. Comparison of bite force and the influencing factors pre- and post-cementation of stainless steel crown in children using T-scan. Int J Clin Pediatr Dent. (2021) 14(1):46–50. 10.5005/jp-journals-10005-1900 34326583 PMC8311754

[38] Ang SF Schulz A Pacher Fernandes R Schneider GA. Sub-10-micrometer toughening and crack tip toughness of dental enamel. J Mech Behav Biomed Mater. (2011) 4(3):423–32. 10.1016/j.jmbbm.2010.12.003 21316630

[39] Zaytsev D Panfilov P. Deformation behavior of human enamel and dentin–enamel junction under compression. Mater Sci Eng C. (2014) 34:15–21. 10.1016/j.msec.2013.10.009 24268228

[40] Thompson VP Silva NRFA. Structure and properties of enamel and dentin. In: Vallittu P, editor. Non-Metallic Biomaterials for Tooth Repair and Replacement. Philadelphia, United-States: Woodhead Publishing (2013). p. 3–19. (Woodhead Publishing Series in Biomaterials).

[41] Ziskind D Hasday M Cohen SR Wagner HD. Young’s modulus of peritubular and intertubular human dentin by nano-indentation tests. J Struct Biol. (2011) 174(1):23–30. 10.1016/j.jsb.2010.09.010 20850543

[42] Celik HK Koc S Kustarci A Rennie AEW. A literature review on the linear elastic material properties assigned in finite element analyses in dental research. Mater Today Commun. (2022) 30:103087. 10.1016/j.mtcomm.2021.103087

[43] Trivedi S. Finite element analysis: a boon to dentistry. J Oral Biol Craniofac Res. (2014) 4(3):200–3. 10.1016/j.jobcr.2014.11.008 25737944 PMC4306993

[44] Minch L. Material properties of periodontal ligaments. Postepy Hig Med Dosw. (2013) 67:1261–4. 10.5604/17322693.1079820 24379266

[45] Saini H Ackland DC Gong L Cheng RO. Occlusal load modelling significantly impacts the predicted tooth stress response during biting: a simulation study. Comp Meth Biomech Biomed Eng. (2020) 23(7):261–70. 10.1080/10255842.2020.1711886 31965827

[46] Jansen van Vuuren L Jansen van Vuuren WA Broadbent JM Duncan WJ Waddell JN. Development of a bite force transducer for measuring maximum voluntary bite forces between individual opposing tooth surfaces. J Mech Behav Biomed Mater. (2020) 109:103846. 10.1016/j.jmbbm.2020.103846 32543410

[47] Revilla-León M Kois DE Zeitler JM Att W Kois JC. An overview of the digital occlusion technologies: intraoral scanners, jaw tracking systems, and computerized occlusal analysis devices. J Esthet Restor Dent. (2023) 35(5):735–44. 10.1111/jerd.13044 37021739

[48] Benazzi S Nguyen HN Kullmer O Kupczik K. Dynamic modelling of tooth deformation using occlusal kinematics and finite element analysis. Papaccio G, éditeur. PLoS One. (2016) 11(3):e0152663. 10.1371/journal.pone.0152663 27031836 PMC4816422

[49] Röhrle O Saini H Lee PVS Ackland DC. A novel computational method to determine subject-specific bite force and occlusal loading during mastication. Comp Meth Biomech Biomed Eng. (2018) 21(6):453–60. 10.1080/10255842.2018.1479744 30010417

[50] Benazzi S Kullmer O Grosse IR Weber GW. Using occlusal wear information and finite element analysis to investigate stress distributions in human molars: occlusal load simulations in molars. J Anat. (2011) 219(3):259–72. 10.1111/j.1469-7580.2011.01396.x 21615398 PMC3171771

[51] Jansen van Vuuren L Jansen van Vuuren WA Broadbent JM Duncan WJ Waddell JN. Development of a bite force transducer for measuring maximum voluntary bite forces between individual opposing tooth surfaces. J Mech Behav Biomed Mater. (2020) 109:103846. 10.1016/j.jmbbm.2020.103846 32543410

